# Service Changes, Utilization, and Financial Performance After Critical Access Hospitals Join Hospital Systems

**DOI:** 10.7759/cureus.82619

**Published:** 2025-04-20

**Authors:** Cody L Mullens, Mitchell Mead, Sean M O'Neill, Calista M Harbaugh, Andrew M Ibrahim

**Affiliations:** 1 Department of Surgery, University of Michigan, Ann Arbor, USA

**Keywords:** critical access hospital, healthcare network, healthcare system, hospital closure, hospital network, hospital system, rural, rural hospital closure

## Abstract

Introduction: Rural hospitals continue to close at unprecedented rates. Joining a hospital system is one strategy some hospitals have taken to avert closure. The objective of this study was to evaluate changes in services, utilization, and financial performance after a critical access hospital (CAH) joins a hospital system.

Methods: This retrospective cohort study used American Hospital Association Annual Survey data and Centers for Medicare and Medicaid Services Provider of Services and Cost Reports data to evaluate changes after a CAH joined a hospital system between 2011 and 2021. To identify whether service provision by CAHs changes following hospital system affiliation, the proportion of CAHs offering each service before and after joining a system was evaluated. A multivariable, interrupted time series model was utilized to investigate changes in utilization and financial profitability up to five years prior to and after joining a system.

Results: A total of 202 CAHs joining a system during the study period were evaluated. Minimal changes in the types of services offered were identified following system affiliation. Specifically, among the 36 different healthcare services evaluated, 34 did not undergo significant changes upon network membership. After joining a hospital system, there was a reduction in the proportion of Medicaid discharges (7.4% vs. 8.2%, p<0.05), Medicare discharges (51.3% vs. 53.6%, p<0.001), average daily census (15 vs. 18, p<0.05), inpatient days (5,447 vs. 6,346, p<0.05), and total facility admissions (555 vs. 649, p<0.01) at CAHs. An increase of 1.3% in CAH profitability (p<0.001) was observed following hospital system affiliation.

Conclusion: While CAHs appear not to change the services provided after joining a hospital system, a reduction in inpatient utilization and an increase in financial profitability were observed. These findings have important implications for healthcare leaders and policymakers interested in ensuring rural communities maintain access to healthcare.

## Introduction

Approximately 80 million Americans rely on critical access hospitals (CAHs) as their primary source of healthcare services [1]. These facilities serve as an essential lifeline for accessing necessary healthcare resources for rural patient populations. The CAH designation was created in 1997 to provide small, relatively isolated hospitals with federal subsidies to improve their financial viability and, in turn, facilitate access to care for rural patients [2,3]. Despite these efforts, rural hospital closures continue to occur at an unprecedented rate, with more than 150 closures since 2010, leading to growing concerns about the timeliness of care for rural patients [4-7]. One strategy to avoid closure that some CAHs have attempted has been to join a hospital system [8].

The impact of CAHs joining hospital systems on healthcare services offered, healthcare utilization, and changes in financial performance remains unexplored. On one hand, after joining a system, CAHs may expand some services after obtaining resources, incorporating policies, and implementing workflows of the hospital system that acquired them. On the other hand, the system may see the CAH as an opportunity to scale down low-profit services while maintaining more profitable services in order to improve the financial viability of a newly acquired hospital. The impact of these mergers on hospital services and profitability for small rural hospitals in general remains less understood [9-13]. Thus, clearer evidence is needed to inform healthcare leaders and policymakers when considering additional strategies to preserve access to healthcare in rural communities [14].

The aims of this study were to assess changes in healthcare services, utilization, and financial performance among CAHs before versus after joining a hospital system. This was accomplished through the use of data from the Centers for Medicare and Medicaid Services (CMS) and the American Hospital Association (AHA).

## Materials and methods

Data source and study population

Data were collected from three large, national longitudinal datasets to inform this retrospective cohort study between 2011 and 2021. First, the CMS Provider of Services file was utilized to identify the hospital cohort and the services provided by hospitals [15]. The CMS Provider of Services files capture all CMS-certified hospitals within the US and are regularly used to evaluate hospital performance. All hospitals designated as CAHs were identified from the Provider of Services file. Additionally, the Provider of Services Files contain data on whether a hospital does or does not provide services for a variety of service lines. These binary data provided an opportunity to evaluate changes in service lines at the hospital level after CAHs joined a hospital system.

Second, the AHA Annual Survey was used to identify whether a CAH was a member of a hospital system during a given year [16]. This database is one of the most comprehensive resources regarding hospital and hospital system data and is regularly treated as a census of hospitals within the United States. While other datasets exist pertaining to hospital mergers and acquisitions, the AHA Annual Survey contains the most detailed and longitudinal dataset on hospital system membership. The year a CAH joined a hospital system was determined by identifying the first year a hospital reported being a member of a system when previously independent. Hospitals that reported switching from independent to a system member multiple times were excluded from this analysis. Additional hospital-level data, including utilization, beds available, and staffing, were also collected from the AHA Annual Survey. Multiple years of AHA data (2011-2021) were included to ensure a sufficient timeframe to detect potential changes after a CAH joined a system.

Finally, hospital financial performance data were collected from the CMS Cost Reports and linked to individual CAHs through their Medicare Provider Identification Number. CMS provides these data through the Hospital Cost Reporting Information System (HCRIS), an annual financial statement required to be reported for all Medicare-certified institutions [17]. This database is publicly available and provides multiple financial variables, including revenue, operating expenses, liabilities, and discharges by insurance type. Financial performance data were collected from 2011 to 2021. Data prior to 2010 were not included due to changes in reporting frameworks implemented by CMS in 2010 [18]. These data were used to obtain an understanding of changes in financial performance prior to and after joining a hospital system. Our final analytic sample included hospitals that had all years of data within the CMS and AHA data files for the time period. Additionally, hospitals missing only one year of data during the study period were still included. For years with missing data, the average between the trailing and following year was used for continuous variables, while the trailing year’s data were utilized for binary variables (e.g., service line variables), as these data are relatively consistent over time and generally not subject to large year-to-year variability. This study was considered exempt from the University of Michigan Institutional Review Board.

Study variables

First, hospital characteristics were compared across CAHs based on hospital system membership status. Variables within the CMS Provider of Services data, such as bed size, ownership, region, and rurality, were evaluated to describe similarities and differences between CAHs and hospital system membership. To broadly understand changes in CAHs after joining a system, multiple variables collected by the CMS Provider of Services and Cost Reports were evaluated. The Provider of Services data identifies 36 healthcare services (Table 1) as binary variables, all of which were evaluated and compared at CAHs before and after joining a system.

Second, service utilization was studied to detect potential changes in the volume of services provided by CAHs between the pre- and post-periods. Service utilization variables included in the AHA Annual Survey include average daily census, total facility admissions, total annual surgeries, total outpatient surgeries, total inpatient surgeries, total inpatient days, total emergency admissions, total births, total outpatient visits, full-time and part-time physicians/dentists, and total facility beds set up and staffed. Each of these variables was compared prior to and after joining a system. The CMS Cost Reports were also utilized to compare the total number of discharges, Medicare discharges, and Medicaid discharges before and after CAHs joined a hospital system. In addition to patient case mix data, changes in patient use intensity were evaluated through the number of inpatient days and outpatient encounters, categorized by payer type. This provided an indicator, based on insurance type, for the relative population of patients receiving care at CAHs before and after joining a system.

Finally, several variables from the CMS Cost Reports were used to measure organizational and financial outcomes of CAHs after joining a system. These measures included total patient revenue, inpatient revenue, total operating expense, net income, operating margin, and total margin, all of which were assessed. The operating margin was calculated as the net income from patient care divided by the total revenue [19]. In contrast, the total margin was income from all sources, including patient care, divided by total revenue. These two variables are commonly used as wide-ranging measures to describe the financial performance of hospitals and inform decision-making. Both operating and total margins were evaluated in the analysis to identify how different sources of income for CAHs may change after joining a system.

Statistical analysis

Our overall analysis had two key goals. First, the aim was to identify changes in healthcare services for CAHs after joining a system. This analysis compared healthcare service offerings one year prior to joining a system to any point five years following joining a hospital system. A five-year post-period was utilized to account for the implementation time of services after joining a system. Second, changes in service utilization and financial outcomes were evaluated for CAHs that joined a system during the study period. The year a CAH joined a system was excluded from the analysis to account for changes that may occur during the year the hospital joined the system. Due to significant skew in hospital financial data, 5% winsorization was applied to the financial data for each calendar year to control for outliers. A multivariable interrupted time-series regression was utilized to evaluate yearly changes in CAH utilization and financial performance following system affiliation. The pre-post quasi-experimental design accounted for hospital-level variation in the analysis of consolidation. The year was included in the model to account for secular trends. While autocorrelation was not explicitly evaluated, robust standard errors clustered at the hospital level were used, as the data were relatively highly aggregated and standardized. Equality of proportions tests and t-tests were applied where appropriate to compare changes between pre- and post-consolidation periods for service line changes (Table 1), care utilization (Table 2), case mix (Table 3), and financial performance (Table 4). All p-values were reported as two-sided with 0.05 as the threshold for significance. Statistical analyses were performed using STATA16 for Windows (StataCorp. 2019. Stata Statistical Software: Release 16. College Station, TX: StataCorp LLC).

## Results

A total of 1,399 hospitals designated as CAHs in HCRIS during the study period were initially identified, with 1,383 hospitals reporting both revenue and operating expenses for at least one year. Additionally, 1,135 hospitals were identified as reporting both HCRIS and AHA data for at least 10 years. After excluding hospitals that switched system membership status multiple times, which may bias our data, or did not join a system during the study period, our final analytic sample was comprised of 202 CAHs that joined a system during the study period.

Table 1 illustrates service line availabilities for CAHs included in the study and compares service line availability changes before and after CAHs joined hospital systems. For most service lines, there were no statistically significant changes in service line availability after CAHs joined a system. Statistically significant increases were identified in the availability of emergency psychiatric services (23% to 39%, p<0.01), outpatient rehabilitation services (59% to 73%, p<0.05), and outpatient psychiatric services (14% to 25%, p<0.05).

**Table 1:** Service line changes after critical access hospitals join hospital systems Authors' Analysis of the CMS Provider of Services File and American Hospital Association Annual Survey 2011-2021. Note: The columns display the percentage of critical access hospitals providing a service line (reported in the CMS Provider Files) by system membership (within the AHA survey). p-values are based on equality of proportions tests for each variable, comparing service availability prior to and after joining a hospital system among 202 hospitals that joined a system during the study period. CT: computed tomography; MRI: magnetic resonance imaging; ICU: intensive care unit; CMS: Centers for Medicare and Medicaid Services; AHA: American Hospital Association

Service line	Before joining a system, n (%)	After joining a system, n (%)	Difference	p-value
Emergency psychiatric services	46 (23%)	79 (39%)	16%	< 0.01
Outpatient rehabilitation services	119 (59%)	147 (73%)	14%	< 0.05
Outpatient psychiatric services	28 (14%)	51 (25%)	11%	< 0.05
Occupational therapy services	170 (84%)	184 (91%)	7%	0.09
Dental services	75 (37%)	87 (43%)	6%	0.34
Orthopedic surgery services	111 (55%)	123 (61%)	6%	0.4
Reconstructive surgery services	18 (9%)	30 (15%)	6%	0.16
CT scan services	170 (84%)	180 (89%)	5%	0.2
MRI services	145 (72%)	156 (77%)	5%	0.33
Speech pathology services	172 (85%)	182 (90%)	5%	0.25
Audiology services	28 (14%)	36 (18%)	4%	0.37
Geriatric psychiatric services	26 (13%)	34 (17%)	4%	0.36
Psychiatric child and adolescent services	14 (7%)	22 (11%)	4%	0.36
Social services	182 (90%)	190 (94%)	4%	0.24
Pediatric services	111 (55%)	117 (58%)	3%	0.65
Respiratory care services	180 (89%)	186 (92%)	3%	0.53
Chemotherapy services	59 (29%)	63 (31%)	2%	0.75
Inpatient surgical services	156 (77%)	160 (79%)	2%	0.78
Neurosurgical services	6 (3%)	10 (5%)	2%	0.51
Outpatient surgery services	162 (80%)	166 (82%)	2%	0.65
Anesthesia services	172 (85%)	174 (86%)	1%	0.88
Clinical laboratory services	200 (99%)	202 (100%)	1%	0.32
Diagnostic radiology services	200 (99%)	202 (100%)	1%	0.32
Physical therapy services	200 (99%)	202 (100%)	1%	0.32
Acute renal dialysis services	6 (3%)	6 (3%)	0%	0.99
Burn care unit services	4 (2%)	4 (2%)	0%	0.99
Cardiac thoracic surgery services	2 (1%)	2 (1%)	0%	1
Emergency department services	198 (98%)	198 (98%)	0%	0.66
Outpatient services	198 (98%)	198 (98%)	0%	0.99
Nuclear medicine services	135 (67%)	133 (66%)	-1%	0.75
Ophthalmic surgery services	81 (40%)	79 (39%)	-1%	0.94
Pediatric ICU services	16 (8%)	14 (7%)	-1%	0.64
Adult inpatient psychiatric services	24 (12%)	20 (10%)	-2%	0.56
Coronary care unit services	36 (18%)	32 (16%)	-2%	0.63
Obstetrics services	79 (39%)	71 (35%)	-4%	0.54
Medical-surgical ICU services	83 (41%)	71 (35%)	-6%	0.39

Changes in the utilization of healthcare services at CAHs before and after consolidation into a hospital system were also compared (Table 2). Average ICU beds (1.01 to 0.83, p<0.05), average daily census (18 to 15, p<0.05), total facility admissions (649 to 555, p<0.01), total facility inpatient days (6,346 to 5,447, p<0.05), and total emergency admissions (6,494 to 5,856, p<0.01) all significantly decreased after CAHs joined a hospital system.

**Table 2:** Changes in healthcare utilization at critical access hospitals after joining a hospital system Authors' Analysis of the CMS Provider of Services File and American Hospital Association Annual Survey 2011-2021. Note: This comparison shows differences in service utilization from the AHA Annual Survey. p-values are based on t-tests to compare data points prior to and following CAHs joining a system. CAH: critical access hospital; AHA: American Hospital Association; CMS: Centers for Medicare and Medicaid Services

Service	Before joining a system, n (%)	After joining a system, n (%)	Difference (%)	p-Value
Inpatient utilization				
Total medical/surgical ICU beds	1.01 (0.1)	0.83 (0.1)	-0.2 (-18%)	< 0.05
Average daily census	18 (2)	15 (2)	-3 (-15%)	< 0.05
Total facility admissions	649 (36)	555 (33)	-94 (-14%)	< 0.01
Total facility inpatient days	6,346 (767)	5,447 (655)	-899 (-14%)	< 0.05
Total births	51 (9)	45 (8)	-6 (-11%)	0.13
Total inpatient surgeries	139 (16)	125 (14)	-14 (-10%)	0.14
Total emergency admissions	6,494 (396)	5,856 (335)	-638 (-10%)	< 0.01
Total staffed beds	35 (2)	32 (2)	-3 (-7%)	0.06
Total annual surgeries	941 (85)	937 (78)	-4 (0%)	0.94
Outpatient utilization				
Total outpatient surgeries	802 (71)	813 (67)	10 (1%)	0.82
Total outpatient visits	38,683 (2,641)	40,909 (3,054)	2,226 (6%)	0.22
Workforce changes				
Full-time physician/dentist	4.05 (0.4)	4.65 (0.6)	0.6 (15%)	0.09
Part-time physician/dentist	1.34 (0.2)	1.66 (0.2)	0.3 (24%)	< 0.01

Payer mixes at CAHs that were never in a system or always in a system were then assessed, and payer mix changes pre- and post-consolidation were compared among the hospitals that joined a system (Table 3). Significant decreases in discharges, inpatient days, and outpatient visits were generally observed across all payers; however, there were significant decreases in the proportion of Medicare and Medicaid patients discharged at CAHs, as demonstrated in Table 3.

**Table 3:** Changes in case mix at critical access hospitals after joining a system Authors' Analysis of the CMS Provider of Services File and American Hospital Association Annual Survey 2011-2021. Note: This comparison shows differences in patient case mix and other variables related to the CMS Cost Reports. p-values are based on t-tests to compare data points prior to and following CAHs joining a system. SE: standard error; CAH: critical access hospital; CMS: Centers for Medicare and Medicaid Services

Service line	Before joining a system, mean (SE)	After joining a system, mean (SE)	Difference	p-value
Medicare discharges	251 (14)	201 (12)	-50	< 0.001
Medicaid discharges	47 (4)	36 (4)	-11	< 0.001
Total discharges	521 (32)	446 (30)	-75	< 0.001
Medicare discharges, % (SE%)	53.6% (1.2%)	51.3% (1.35%)	-2.30%	< 0.001
Medicaid discharges, % (SE%)	8.2% (0.5%)	7.4% (0.58%)	-0.70%	< 0.05
Other discharges, % (SE%)	38% (1.09%)	41.1% (1.3%)	3%	< 0.001
Medicare inpatient days and outpatient visits	813 (45)	634 (39)	-179	< 0.001
Medicaid inpatient days and outpatient visits	115 (10)	89 (9)	-26	< 0.001
Total inpatient days and outpatient visits	1,476 (88)	1,250 (82)	-226	< 0.001
Medicare inpatient days, % (SE%)	59% (1.15%)	55.6% (1.21%)	-3.40%	< 0.001
Medicaid inpatient days, % (SE%)	7.2% (0.41%)	7% (0.51%)	-0.30%	0.38
Other inpatient days, % (SE%)	34% (1.07%)	38.5% (1.16%)	4.50%	< 0.001

**Figure 1:**
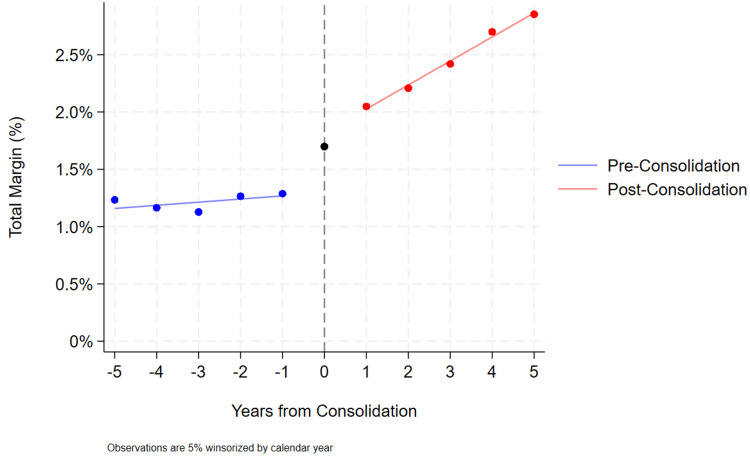
Changes in total margin before and after critical access hospitals join a hospital system

Finally, the financial performance of CAHs was analyzed and compared based on hospital system membership (Table 4). While inpatient revenue significantly decreased, outpatient revenue increased, with an overall significant improvement in total revenue after consolidation, despite operating expenses also increasing. Other markers of financial performance also suggested post-consolidation improvement, including increases in cash on hand and total margin, while bad debt decreased. Figure 1 demonstrates the changes in hospital margin among CAHs prior to consolidation and after consolidation.

**Table 4:** Financial performance at critical access hospitals after joining a system Authors' Analysis of the CMS Provider of Services File and American Hospital Association Annual Survey 2011-2021. Note: This comparison shows differences in case mix from the CMS Cost Reports. The analysis compares the five years before and after the consolidation year while excluding the year of consolidation. All data are 5% winsorized by year to account for outliers. The model includes the calendar year to account for secular trends. To account for the correlation of data within hospitals, standard errors are clustered at the hospital level. p-values are based on the results of equality of proportions tests and t-tests, where appropriate. CC: cost-to-charge ratio; CMS: Centers for Medicare and Medicaid Services

Financial measure	Pre-consolidation adjusted mean (SE)	Post-consolidation adjusted mean (SE)	Difference (% change)	p-value
Total revenue, $ million	47.9 (3.1)	56.4 (3.7)	8.5 (17.7%)	< 0.001
Total operating expense, $ million	24.5 (1.4)	27.6 (1.5)	3.1 (12.7%)	< 0.001
Inpatient revenue (% of total revenue)	25% (0.9%)	22% (1%)	-3%	< 0.001
Outpatient revenue (% of total revenue)	75% (0.9%)	78% (1%)	3%	< 0.001
Other income (% of total income)	5% (0.4%)	6.4% (0.5%)	1.40%	< 0.001
Uncompensated care, CC adjusted (% of total operating expense)	4.5% (0.3%)	4.6% (0.2%)	0.10%	0.48
Bad debt (% of operating expense)	7.9% (0.5%)	6.4% (0.4%)	-1.50%	< 0.001
Operating margin	-4.1% (0.6%)	-4.3% (0.6%)	-0.20%	0.34
Total margin	1.1% (0.3%)	2.4% (0.3%)	1.30%	< 0.001
Cash on hand, $ million	3.3 (0.3)	5.8 (0.5)	2.5 (75.8%)	< 0.001
Days cash on hand	47.4 (3.7)	76.3 (5.7)	28.9 (61%)	< 0.001

## Discussion

This evaluation of changes in healthcare services, utilization, and profitability after CAHs join a hospital system had three principal findings. First, it was observed that service lines appear to be maintained after previously independent CAHs joined a hospital system. Second, there was a significant decrease in inpatient utilization (e.g., daily census, facility admissions, and staffed beds). Third, an improvement in the profitability of CAHs was found after joining a hospital system. Taken together, while CAHs that join hospital systems maintain service availability after consolidation, profitability improves in the setting of scaled-back healthcare resource utilization at these hospitals.

The impact that CAH system membership has on service availability and profitability has been explored previously. When rural hospitals merge, there is evidence that these hospitals are more likely to eliminate neonatal, maternal, surgical, and behavioral health care compared to independent rural hospital counterparts [20]. Other evidence on rural hospital mergers has found that becoming affiliated with a hospital system is associated with decreased availability of diagnostic imaging technologies, decreased availability of primary and obstetric care, and decreased outpatient visits, but it has observed an increase in operating margins [12]. Our present study provided additional context for changes in CAHs, a subtype of rural hospitals, after joining a system. Specifically, the availability of nearly all queried services appears unchanged after joining a system. However, utilization of these maintained service lines shifted to the outpatient setting. In this context, our findings suggest heterogeneity in how small rural hospitals change after joining a hospital system.

While our data cannot elucidate the exact mechanism by which CAHs joining a hospital system improves their profit margin, our data suggest at least two possibilities. First, CAH appears to shift utilization to focus less on inpatient services and more on outpatient services. These latter services may be less resource-intensive with less overhead, which makes them more amenable to a viable profit. Second, after joining a hospital system, CAH saw a change in payer mix. Specifically, decreases were noted in Medicare and Medicaid beneficiaries, populations known to receive lower reimbursement than their privately insured counterparts.

Our study should be interpreted in the context of multiple limitations. First, the AHA Annual Survey and CMS Cost Reports are databases derived from annual surveys and forms that are self-reported by hospitals. However, these databases include the largest sample of hospital characteristics, utilization, and financial data and are widely used in health policy research for these purposes. Second, this study solely focuses on changes at the hospital level after joining a system and does not provide information on patient-level clinical outcomes. While this may limit the understanding of CAH system membership and quality of care to patients, this study provides a comprehensive analysis related to service availability, utilization, and financial performance, which were the primary aims of this paper. Moreover, several other prior studies have evaluated the quality of care at CAHs [21-23]. These measures are also relevant factors for hospital performance and can potentially provide evidence for informing strategy for healthcare leaders on hospital system membership and inform policy with respect to CAHs. Finally, other unevaluated variables may influence variation across CAHs. Potential omitted variable bias was mitigated in two ways. First, the year was incorporated into the regression to account for national trends in services over time, which may confound the evaluation of hospital system membership. Second, standard errors were clustered at the hospital level to adjust for the correlation of data within hospitals across the time period.

The findings of this work have implications for a wide range of stakeholders. For the local rural community that is served by a CAH, our findings suggest that CAHs joining a hospital system largely maintain the availability of healthcare services locally within their communities. For community members and local CAH leaders of independent facilities who have concerns about services being siphoned away after joining a system, our data provides an alternative perspective. The subset of CAHs included in this study appears to maintain their breadth of service availability in the setting of improved profitability, which may contribute to longer-standing sustainability of the hospital in the local community. However, while the services appear to remain available, there was a significant decrease in the utilization of these services based on facility admission and encounter numbers.

For healthcare leaders at CAHs with narrow or diminishing profit margins, our findings suggest joining a hospital system may enhance financial viability. While these CAHs’ profitability improved after joining a system, their low pre-consolidation margins allude to the significant financial vulnerability these hospitals were facing at the time of joining a system. System membership may have been an influential strategy to stay open and continue providing healthcare services to local rural communities. These findings may shed important light on this strategy for other rural healthcare leaders who are facing possible closure. Given the recent growth over the decade in the number of CAHs joining a system, joining a system appears to be an important strategy for some CAHs.

For policymakers focused on improving hospital access for rural patients, they may consider evaluating CAHs differently based on their system participation status. CAH designation provides significant financial benefits because of providing services in rural communities, which often lack privately insured patients and provide greater amounts of uncompensated care. It was found that CAHs that joined a system served a higher proportion of patients outside of federally insured plans. This suggests that hospitals that are versus are not part of a hospital system appear to differ by patient case mix and financial performance, all of which should be taken into consideration when evaluating access to services for rural communities.

Finally, a new CMS policy was enacted in 2023 that enables CAHs to receive federal subsidies in exchange for eliminating inpatient services and designating themselves as rural emergency hospitals [24,25]. The aim of this policy is to help CAHs avert closure by transitioning the care they provide to centering on emergency and outpatient care. There are growing concerns around the potential unintended consequences of this policy, especially with respect to accessing inpatient care [26,27]. Even prior to the enactment of this policy, trends in this study suggested that CAHs joining a system reduce inpatient care, despite evidence of continued service availability in these settings. The data presented here suggest a potential alternative strategy for CAH to avert closure outside of the rural emergency hospital program. These findings may also have particular relevance for potential future policies, including considerations of site-neutral payments and other recently introduced federal legislation.

## Conclusions

It was found that, after joining a hospital system, CAHs can improve profitability, despite maintaining service availability and decreasing the utilization of inpatient services. These findings have important implications for rural communities, hospital leaders, and policymakers as new CAH policies are implemented and future interventions are developed. Future work should evaluate the long-term impacts of CAHs joining hospital systems, with careful attention to monitoring for unintended consequences of joining a system for both small rural hospitals and the patients they serve.
